# Analysis of Heat Treatment of AlSi7Cu0.5Mg Alloy

**DOI:** 10.3390/ma18040733

**Published:** 2025-02-07

**Authors:** Miloš Mičian, Elena Kantoríková, Joanna Borowiecka-Jamrozek, Justyna Kasińska

**Affiliations:** 1 Faculty of Mechanical Engineering, University of Zilina, Univerzitná 8215/1, 010 26 Žilina, Slovakia; elena.kantorikova@fstroj.uniza.sk; 2 Faculty of Mechatronics and Mechanical Engineering, Kielce University of Technology, 25-314 Kielce, Poland; jamrozek@tu.kielce.pl (J.B.-J.); kasinska@tu.kielce.pl (J.K.)

**Keywords:** heat treatment, aluminum alloys, mechanical properties, automotive

## Abstract

This article describes how different heat treatment methods affect the mechanical properties of AlSi7Cu0.5Mg. This article offers a new perspective on the topic of heat treatment analysis. AlSi7Cu0.5Mg alloy has a specific chemical composition due to its lower titanium and silicon content. It is still an experimentally tested alloy, intended for the production of high-strength castings in automotive applications. Although we are generally familiar with heat treatment methods for aluminum alloys, in the present case, we did not know the exact effect of artificial aging on the mechanical properties of AlSi7Cu0.5Mg. Five heat treatment regimes at temperatures of 160; 180; 200; 220 and 240 °C were monitored by removing the samples from the furnace at specified time intervals ranging from 30 min to 8 h. The solution treatment was the same for all samples (540 °C, 4 h and 80 °C water). The aim was to find out at what the highest hardness, strength, ductility, yield strength and metallographic yield strength were which could be obtained. The best properties were presented by the alloy which was artificially aged at 180 °C for 60 min. The topic and research on aluminum alloys in general are still relevant because in industrial practice and automotive applications, Al alloys are irreplaceable.

## 1. Introduction

Aluminum alloys have a wide range of industrial applications and are suitable to be processed by a various technologies. Their great advantages are their high corrosion resistance, dimensional stability at different working temperatures and good mechanical properties. Cast aluminum alloys are characterized, among other things, by their relatively low casting temperature, good castability, easy mold filling and low specific gravity. Among the mechanical properties are the hardness, which is increased primarily by heat treatment, and strength due to the density-to-strength ratio of these alloys. The mechanical properties are, of course, modified by the purity of the aluminum, the silicon content and other additive elements. The processing technology and the parameters set in the process also have a significant influence on the properties. Research is still being carried out regarding innovations in both materials and technology. The future of aluminum alloys depends on further developments, especially in production and heat treatment technology [1]. In practice, casting, alloying and heat treatment technology must be combined, as all of these affect the structure of the material and thus its potential. The heat treatment of aluminum alloys is often neglected, or alloys which do not need to be heat treated are preferentially used. It is understandable to use lighter and economically advantageous methods. However, it is currently the case for aluminum alloys intended for the electro-automotive industry that time and energy must also be devoted to heat treatment of the material. Cu and Mg are the most common alloying elements for Al-Si alloys [1,2,3]. Al-Si-Mg-based alloys are characterized by a magnesium content of 0.25–0.6 wt.%, which is precipitated at normal cooling rates as the Mg_2_Si phase. In the cast state, their properties are average [4]. The Mg_2_Si phase allows precipitation hardening such that they are heat-treatable (hardenable) alloys, which subsequently achieve high mechanical properties. As the Si content increases, the foundry properties and weldability improve, and the content of accompanying elements is limited to extremely low values [5,6]. Al-Si-Cu-based alloys are predominantly sub-eutectic and, in exceptional cases, eutectic. Thus, the Si content ranges from 6 to 13 wt.%. Copper is present in amounts ranging from 1 to 5 wt.%. If the Cu content is sufficiently high, then stable β-Mg_2_Si, θ-Al_2_(Al,Cu) phases are formed in the Al-Si-Mg-Cu system. Fe-containing phases and a eutectic Si phase are formed during solidification and cooling [7,8]. Al-Si-Cu-based alloys are heat treatable through hardening, and this is due to the variable solubility of Cu in Si and formation of the intermetallic compound Al_2_Cu. The copper in these alloys even allows so-called natural or spontaneous curing, where precipitation hardening occurs without the need to raise the casting temperature [9,10,11]. This process takes many times longer than elevated-temperature curing and does not achieve such high values, but the increase in the mechanical properties is noticeable. The properties of these alloys vary with the Cu and Si contents. With an increasing Cu content, the mechanical properties increase, but the corrosion resistance and plastic properties deteriorate [12]. The presence of Zn and Mg further increases this effect. Copper also positively influences the machinability and foundry properties, such as the shrinkage or pressure tightness of castings. There is also less tearing and withdrawal in these alloys, and it is therefore advantageous to keep the Si content as high as possible. The treatability also increases with an increasing Si content [13,14]. Lower silicon (Si) and titanium (Ti) contents can affect the aging behavior of an alloy. Silicon and titanium are elements which can improve the strength and toughness of alloys, especially during heat treatment such as aging. Reducing their contents can lead to changes in the microstructure of an alloy, which can affect mechanical properties such as its strength, hardness and corrosion resistance. Lower silicon contents can lead to a decrease in strength and hardness, as silicon contributes to the formation of hard phases in the microstructure. Lower contents of these elements can affect heat treatment processes such as aging and thus the final mechanical properties of an alloy. The important elements which affect the heat treatment of aluminum alloys are silicon, copper and magnesium. Silicon is the main alloying element for binary Al-Si alloys and is usually used in concentrations from 5% to 25%. Together with Al, they form the binary eutectic α(Al)+Si, where α(Al) is the substitutional solid solution of Si in Al. Thus, the eutectic is a mixture of a substitutional solid solution of α(Al) and crystals of nearly pure Si. The silicon particles appear in the microstructure as dark particles distributed in a light matrix α. The amount, shape, size and distribution of Si are highly related to the mechanical properties [15]. In addition to the mechanical properties, corrosion resistance, treatability, thermal expansion and the danger of hot tearing are improved with an increasing Si content [16,17]. The addition of copper produces the intermetallic compound Al_2_Cu in Al-Si, which mainly affects the mechanical properties. This makes the alloy heat treatable through hardening, which causes an increase in properties such as the strength and hardness. It also reduces the shrinkage of the alloy and improves machinability. As a negative effect, we can consider the decrease in ductility caused by the increase in strength properties and also its effect on the solidification interval, which expands, increasing the risk of tearing. Copper also impairs corrosion resistance such that its content is strictly limited in, for example, the food industry. In a solid solution, α(Al) copper has limited solubility and can occur in two modifications as Al_2_Cu and Al-CuAl_2_-Si. The Al_2_Cu particles appear in the alloy as small oval grains with a high Cu concentration and Al-CuAl_2_-Si as a ternary eutectic [18,19]. The formation of ternary eutectics is caused by the limited solubility of Cu in the solid solution of α(Al), and at a content above 1 wt.%, its precipitation already occurs. Magnesium is one of the main additives of Al-Si alloys in the amount of 0.3–0.6 wt.%. Its most important role is to increase the strength of these alloys. The presence of magnesium produces the Mg_2_Si phase, which forms tiny skeletal Al-Si-Mg_2_Si formations with a solidification temperature of about 555 °C, and in the presence of copper, magnesium forms phases primarily with it [19]. In the as-cast condition, it does not have a significant effect on the strength gain. It only plays a significant role during heat treatment, specifically hardening, which is made possible by it. As the Mg content increases, the strength of the alloy also increases, but its influence can also lead to precipitate expulsion at the grain boundaries, which causes susceptibility to intergranular fractures and intergranular corrosion. The strength and hardness of Al-Si-Cu-Mg is increased by the application of heat treatment [20,21,22].

The mechanical properties of heat-treatable alloy components can be optimized by selecting an appropriate solution treatment and aging process. For some alloys, for example, corrosion resistance can be improved at the expense of strength, and vice versa. Solution treatment is normally carried out in the temperature range of 450–575 °C in air, followed by rapid cooling in cold water, hot water, boiling water, a water-polymer (glycol) solution, water spray or forced air flow. Immediately after cooling from the solution treatment, all alloys are relatively soft and can be slightly shaped or straightened within a few hours. These alloys naturally age and harden at room temperature, and their hardness gradually increases after cooling. If the material needs to be shaped or straightened for more than a few hours after cooling, then this phenomenon can be suppressed by cooling to a temperature of about 0 °C. Solution treatment consists of heating to the temperature of maximum solubility of the additive element to allow curing, followed by holding at this temperature and rapid cooling. During annealing at the annealing temperature, all of the segregate is dissolved so that the structure is formed only by the homogeneous α phase. The cooling must be rapid enough to prevent the conditions for reverse segregation. The result is then not a solid solution of α with the hardening phase excluded at the grain boundary but a single-phase, supersaturated solid solution [23,24]. The setting of appropriate solution treatment parameters is highly important. The solution treatment temperature should be above the solubility change curve. In practice, temperatures between 10 and 15 °C below the eutetic temperature are most commonly chosen. The residence time at the dissolution temperature must be long enough to ensure dissolution of all intermetallic phases. As a rule of thumb, this is several hours, and the choice depends on the chemical composition, structure and wall thickness of the casting. With an increasing grain and wall thickness in the castings, this time increases [25]. Cooling from the dissolution temperature is also significant, and certain conditions must be ensured. In addition to rapid cooling to a temperature of 150–200 °C, the time for transferring the casting from the furnace to the cooling tank, which should not exceed 10–20 s, must also be observed. Hot water at 40–80 °C is most commonly used as the cooling medium, and it is recommended to ensure that the water is flowing so that it cools evenly. Failure to comply with some of these conditions may result in expulsion of the initially molten intermetallic phases, which subsequently deteriorates the resulting properties [26].

After the solution treatment, the unstable supersaturated solid solution decays (i.e., ages). The decomposition produces rather small dispersed particles of the secondary phase dispersed in the base matrix. Aging can be divided into natural aging and artificial aging [27]. Natural aging occurs at ambient temperature, unlike artificial aging, but the process takes many times longer. Artificial aging is the decomposition of a supersaturated solid solution at elevated temperatures, most commonly in the range of 150–200 °C, over a period of several hours, depending on the chemical composition of the alloy [28]. Aging is a diffusion process because here, the transition of the additive element to microscopic regions richer in the element in question occurs just by diffusion. This depletes the solid solution of this element and also leads to the formation of precipitates at the sites of clustering of these atoms [29]. The precipitates formed undergo different transition states, and their number may vary from alloy to alloy. Alloys from the Al-Cu system have the largest number of transition states, but in general, the change in the precipitates’ state during the aging process has three phases [30]. The alloys are usually processed to a state where they reach a coherent or semi-coherent phase, when hardening leads to the greatest increase in strength and hardness. However, there are also cases where an incoherent phase is reached, which is the beginning of the decline in hardness and strength in aging. This is known as overaging. In aging, although the strength and hardness decrease, the ductility increases at the same time. For this reason, the alloy is also deliberately heat treated to such a state, which we refer to as the T6 heat treatment state [31]. Thus, in aging, the hardness and strength increase over time up to a certain maximum and then only decrease, and we refer to this decrease as overaging, in which ductility increases. As the temperature increases, the hardening effect diminishes, but the ductility and yield strength increase.

Aluminum alloys have a large presence in the foundry industry, but despite their good foundry properties, in their raw state, they are not always suitable for final consumption. AlSi7Cu0.5Mg alloy has low hardness and strength properties because it consists of eutectic silumin. The structure is composed of a large eutectic β phase on a background of dendritic eutectic α. The mechanical properties can be improved by changing the size and shape of the microstructure components of the alloy. One possibility for influencing the structure is heat treatment. This article is focused on the research on the influence of artificial aging on the mechanical properties of an alloy. The aim was to find out at which parameters the highest hardness, strength, ductility and yield strength can be obtained. The samples were heat treated in the temperature range of 160–240 °C and at different residence times from 60 min to 8 h. The experimental work was time-consuming, as 45 specimens were used for one temperature regime, and thus a total of 230 specimens were used. In this paper, only the average values of the individual measurements were selected. The samples with the highest values for their mechanical properties were also evaluated metallographically. This article offers a new perspective on the topic of heat treatment analysis. It may not belong to the original research of this type, but it provides exclusive results. The topic and research on aluminum alloys in general is still relevant because in industrial practice and automotive applications, Al alloys are irreplaceable. The aim is to extend the knowledge on the effect of artificial aging on AlSi7Cu0.5Mg. The most favourable regime appeared to be in the temperature interval of 180–200 °C, although a high hardness was achieved even after 60 min. The high temperature and short residence time of artificial aging of the aluminum alloy was rather negatively affected. AlSi7Cu0.5Mg is comprehensively one of the most highly used materials in the transport industry, and therefore, experimental works aimed at achieving an increase in mechanical properties via suitable modification of its heat treatment regime are currently becoming more and more widespread.

## 2. Materials and Methods

The experimental material was the aluminum alloy AlSi7Cu0.5Mg, which is currently used for engine head castings. Samples of the AlSi7Cu0.5Mg alloy were cast using gravity casting technology into a detachable metal mold. An electric resistance furnace with a capacity of 15 kg was used for melting. The melt was manually taken directly from the electric resistance furnace and cast into the mold using a steel ladle. Before each casting, the oxide layer was mechanically removed from the surface, thus preventing its formation in the mold cavity. The mold was preheated to 200 °C.

In Table 1, the chemical composition of the experimental alloy measured with an ION2 spectrometer is presented. The standard mechanical properties according to Bayram metal (ENAC-45500) are in Table 2.

**Figure 1:**
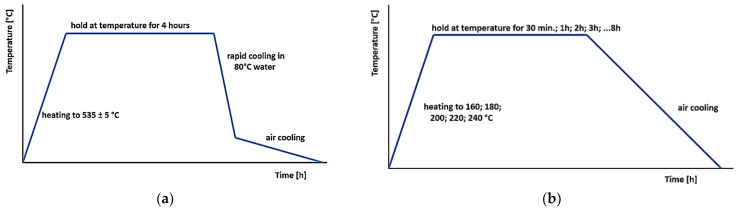
Heat treatment mode used: (**a**) solution treatment and quenching or (**b**) artificial aging.

After the casting process, the samples were prepared for heat treatment. In the first step of heat treatment, all samples were subjected to the solution treatment. The solution treatment temperature was set to 535 °C ± 5 °C. This temperature is commonly used in practice. A higher temperature could lead to local melting of the sample, which would negatively affect the resulting properties. After ramping up to this temperature, the samples were left in the oven for 4 h to ensure a complete solution of all curing inter-metallic phases, particularly the Mg_2_Si phase, as this phase needs more time to dissolve than Al_2_Cu. It was also important to observe the cooling conditions to avoid reverse segregation of the intermetallic phases. Specifically, this involved the time of sample transfer and the temperature of the cooling medium. The time between opening the oven door and immersion of the samples in water must not exceed 20 s. The cooling medium was hot water at 80 °C. After cooling to 100–150 °C, the samples were cooled to ambient temperature in air. The thermal cycle was monitored and recorded using a thermocouple attached to one of the samples. A graphical representation of the solution treatment for the present work is presented in Figure 1a.

In the second step, artificial aging was applied to the experimental samples. After the solution treatment, the samples were divided into 45 groups. The samples were processed at five temperatures with a regular temperature increment of 20 °C, starting at 160 °C and ramping up to 240 °C. The residence time of the samples in the oven was equally graded at each temperature. The shortest residence time for the samples was 30 min after reaching the desired temperature, followed by 1 h, and for each additional sample, the residence time was +1 h up to 8 h. After artificial aging, the specimens were machined and evaluated for their hardness, ultimate strength (Rm), conventional yield strength (Rp0.2), ductility, and microstructures. The artificial aging for a given experiment is presented in Figure 1b. Sample sets were prepared for experimental testing. Each set contained 5 samples.

## 3. Results

### 3.1. Microstructure

A common heat treatment for aluminum alloys is T6, which consists of a dissolution anneal followed by artificial aging. This experimental work presents different methods of artificial aging. Solution treatment increased the ultimate tensile strength and ductility, while aging increased the ultimate tensile strength at the expense of ductility. Heat treatment resulted in the microstructural changes presented. Heat treatment caused the following changes in the microstructure: dissolution of solutes, spherodization of eutectic Si, morphological changes in Mg- and Cu-rich intermetallic phases, recrystallization of eutectic grains and homogenization.

**Figure 2:**
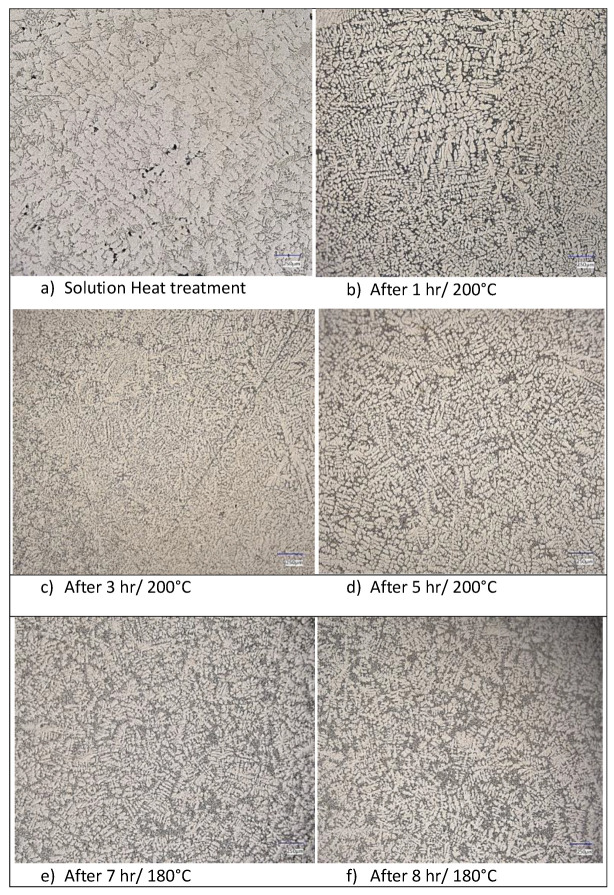
Microstructures of selected alloys etched at 0.5% HF (magnification: 200×).

Before thermal bonding, the structure was formed by α-phase dendrites and rounded silicon grains. Solution treatment affected the microstructure by increasing the content of α-phase dendrites. The structure was homogeneous, and dendrites formed the content of a larger surface. Silicon showed gradual coarsening. With gradual heat treatment, the silicon changed its shape. It manifested itself as an angular and coarse grain, which was scattered at the edge of the α phase. Solution treatment was the starting condition in the given experiment. It is interesting to follow the process of silicon transformation from solution treatment through various modes of artificial aging, which can be observed in Figure 2.

The morphology of silicon in its initial state is rod-shaped, resembling a sea coral. Artificial aging influenced the shapes of the rods. As the so-called coral began to grow, the silicon rods began to separate from each other. Gradually, coarse and rounded grains of sub-eutectic silicon were formed. Spheroidization took place. Here, it is interesting to observe that the spheroidization process stopped at temperatures above 200 °C after only 1 h, but at lower temperatures (below 180 °C), this only occurred after 3 h. The change in the shape of the silicon from plate-like to an oval shape caused a significant increase in the mechanical properties.

Aging causes precipitation of one or more Mg_2_Si or Al_2_Cu phases in Al-Si-Cu-Mg alloys, depending on the chemical composition. These precipitates cause hardening of the alloy. The precipitation enhancement reaction in these alloys is caused by the decomposition of a metastable, supersaturated solid solution obtained through heat treatment and cooling. The phases reported to precipitate during aging of Al-Si-Cu-Mg alloys (depending on the chemical composition) are Al_2_Cu and Mg_2_Si. The Al_2_Cu phase has a preferential tendency to precipitate at dislocations around Si particles. The Al_2_Cu phase contributes significantly to the aging behavior of these alloys because it is uniformly distributed in the Al matrix.

**Figure 3:**
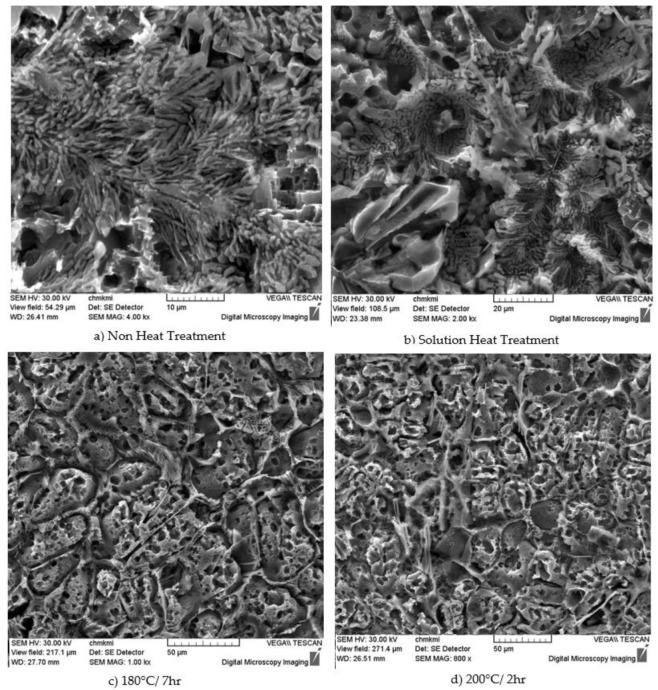
Fractographic evaluation of experimental alloys: (**a**) no heat treatment and (**b**) structure after solution treatment and (**c**,**d**) after artificial aging.

Artificial aging will accelerate the diffusion of dissolved elements and thus ensure the formation of suitable precipitates. The effect of heat treatment is pronounced due to the silicon content. For comparison and a better understanding of the process, Figure 3a shows non-heat-treated AlSi7Cu0.5Mg, where silicon spheroidization has not yet taken place. The silicon particles grow at the expense of the rest (which dissolve), become coarser and acquire a spherical shape. At the same time, they also see increases in hardness and ultimate strength. Under artificial aging, the silicon particles shrink, splitting into smaller segments. The overall structure is finer. The shape of sub-eutectic silicon is approximately the same after artificial aging, and only the processions leading to its formation are different.

Sub-eutectic silicon after fragmentation or branching is formed by thinning from an originally thick plate-like hexagonal silicon crystal. The cooling rate during the solution treatment process was used to achieve spheroidization, growth and coarsening of the silicon grain. At the same time, the number of Si particles per unit area was reduced (Figure 3b). The structures in Figure 3c,d are those after artificial aging. Obviously, the structure was finer and more homogeneous, and the silicon particles were split into smaller ones and obtained a rounded shape.

Samples for transmission electron microscopy (TEM) were prepared using the polishing technique with the Gatan PIPs (Precision Ion Polishing System). The process involved removing material from the sample using an ionized argon beam. The samples were circular with a diameter of 3 mm and a thickness of 70 μm. At the beginning of polishing, an angle of 8° was set, which was gradually reduced to 4° and 2° from both sides of the sample as a hole formed in it. This procedure ensured that the area around the hole was thin enough to observe the material. For TEM, a transparent sample with a thickness of up to 100 nm is required. This method ensured that the edges of the sample remained thicker, while the center was thin enough for observation.

For TEM evaluation, six samples were selected. The samples were aged at the following conditions: 180 °C for 1 h; 180 °C for 7 h; 200 °C for 1 h; 200 °C for 7 h; 220 °C for 0.5 h and 220 °C for 7 h.

**Figure 4:**
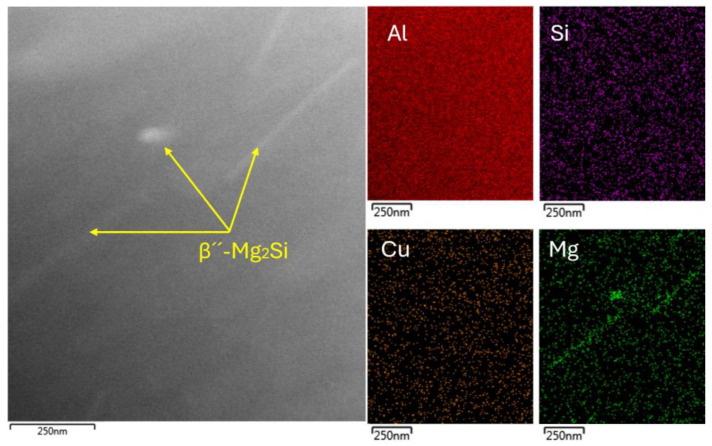
TEM EDS mapping of 180 °C for 1 h sample.

The substructure of the sample aged at 180 °C for one hour is characterized by a smaller number of present precipitates. Energy dispersive spectroscopy (EDS) mapping showed that the rod-like particles were primarily composed of Mg_2_Si (Figure 4). These precipitates were present in the form of thin needles and locally in the form of rounded grains. The lower number of coherent β″-Mg_2_Si precipitates in the substructure was related to the short aging time (1 h) at 180 °C, where the aging process only reached the initial stage of coherent cluster formation.

**Figure 5:**
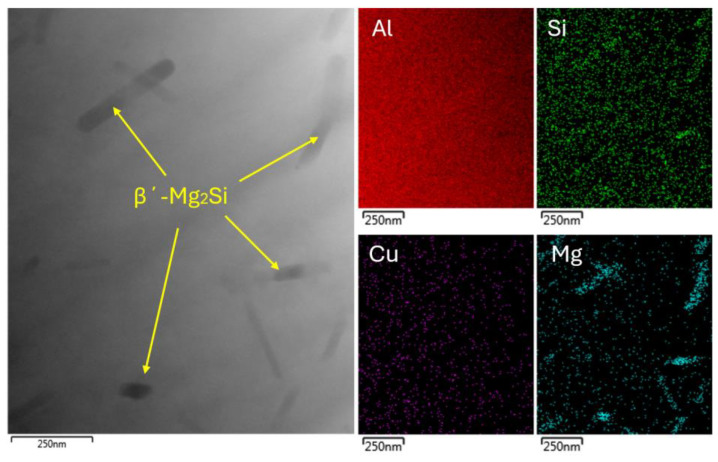
TEM EDS mapping of 180 °C for 7 h sample.

The sample aged at 180 °C for up to 7 h showed ideal properties. An increased number of needle-shaped and rod-shaped particles was present in the substructure (Figure 5). The high number of precipitates in the substructure of the sample indicates that the time of 7 h at 180 °C was sufficient for the formation of coherent precipitates (needle-shaped) and partially coherent precipitates (rod-shaped), but at the same time there, was no so-called overaging and therefore the transformation of coherent or partially coherent precipitates into incoherent particles. Coherent precipitates cause the presence of deformation fields in the matrix and thus prevent and slow down the movement of dislocations, which leads to an increase in strength.

**Figure 6:**
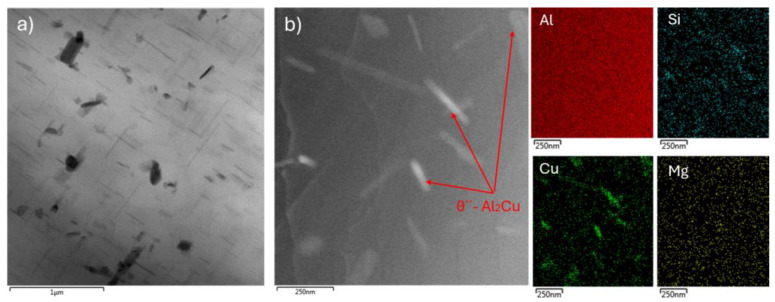
TEM of 180 °C for 1 h sample: (**a**) high annular angular dark field (HAADF) and (**b**) EDS mapping.

Increasing the aging temperature to 200 °C and holding at this temperature for 1 h led to the formation of a large number of particles primarily with a needle-like morphology and a perpendicular orientation to each other (Figure 6). The higher number of precipitates present in the substructure (compared with the 180 °C for 7 h sample) and the needle-like shape indicate that the hardening process at 200 °C and holding for 1 h reached the stage where non-equilibrium coherent precipitates θ″ were formed, which led to the maximum hardness and strength.

**Figure 7:**
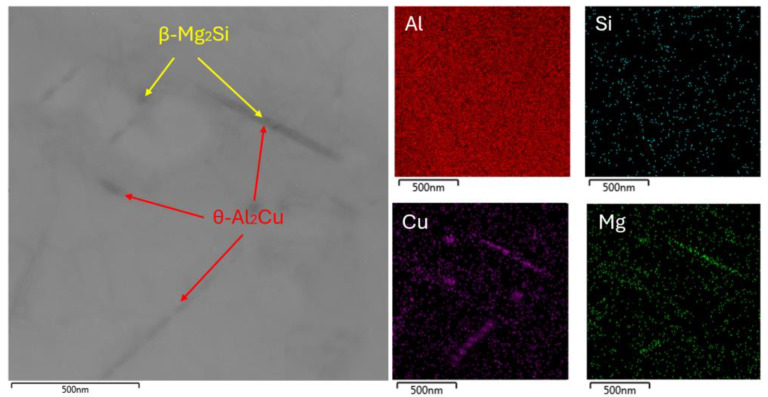
TEM EDS mapping of 200 °C for 7 h sample.

The holding time of 7 h at a temperature of 200 °C, as expected, led to overaging of the alloy. In the substructure of the 200 °C for 7 h sample, a smaller amount of precipitating particles was present (Figure 7). The last stage of aging was accompanied by a decrease in the number of coherent and partially coherent precipitates and their transformation into incoherent particles θ or β, with an increase in their lengths. The incoherent phase no longer had a crystalline bond to the solid solution α, as a result of which the strength properties decreased.

**Figure 8:**
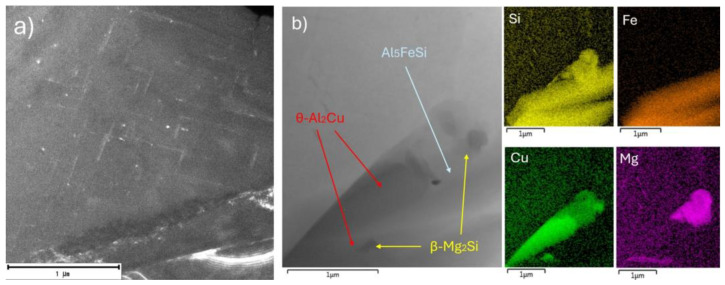
TEM of 180 °C for 1 h sample. (**a**) High annular angular dark field (HAADF) for 220 °C for 0.5 h sample. (**b**) EDS mapping of 220 °C for 7 h sample.

In the substructure of the 220 °C for 0.5 h sample, an increased number of needle-shaped particles oriented toward each other was present (Figure 8a). The presence of these particles clearly indicates that even an extremely short holding time (0.5 h) at a higher temperature (220 °C) was sufficient for the sample to reach the stage of the process of predominantly needle-shaped coherent precipitates forming, which similar to the 200 °C for 1 h sample could maximize the hardness and strength of the alloy.

On the other hand, a 7 h holding time at a temperature of 220 °C, similar to the 200 °C for 7 h sample, led to the formation and presence of incoherent phases in the substructure (Figure 8b). The formed phases of the θ-Al_2_Cu or β-Mg_2_Si type were present in grains with larger dimensions near the Fe-based phase AlFe_5_Si.

**Figure 9:**
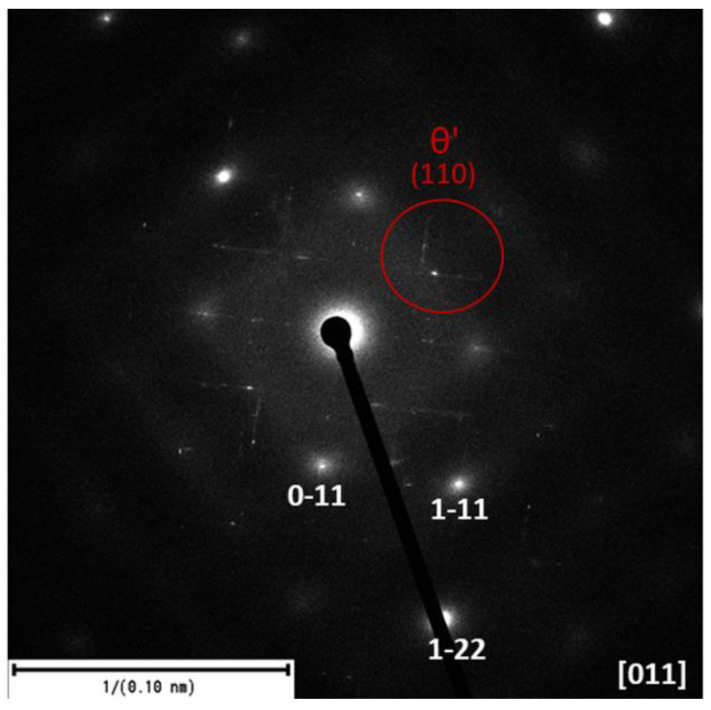
Diffractogram of 180 °C for 1 h sample with different camera distances.

The selected area electron diffraction (SAED) experimental technique is part of a transmission electron microscope. SAED provides information about the crystallography and orientation of crystalline materials (Figure 9). In the SAED image, the regularity of the maxima (lines) of the precipitates θ′ can be observed, which indicates their regular orientation relative to the matrix and is precisely crystallographically determined.

### 3.2. Hardness

For the Brinell hardness test, a sintered carbide ball 5 mm in diameter was used. The loading force was 250 kN for a period of 10 s. The number of indentations on each specimen was seven, from which the average value was calculated. The impressions were distributed over the entire cross-section, capturing both the peripheral and central portions of the me-wound specimen. The diameter of the impression was manually marked, and therefore the values may have had slight variation due to the fact that this was an individual evaluation of the impressions [32]. All measured average values were processed in Table 3 according to the temperatures at which the artificial aging was pre-run, where DA denotes the state after the solution treatment. The tables present the highest and lowest measured hardness values for the different artificial aging regimes.

**Figure 10:**
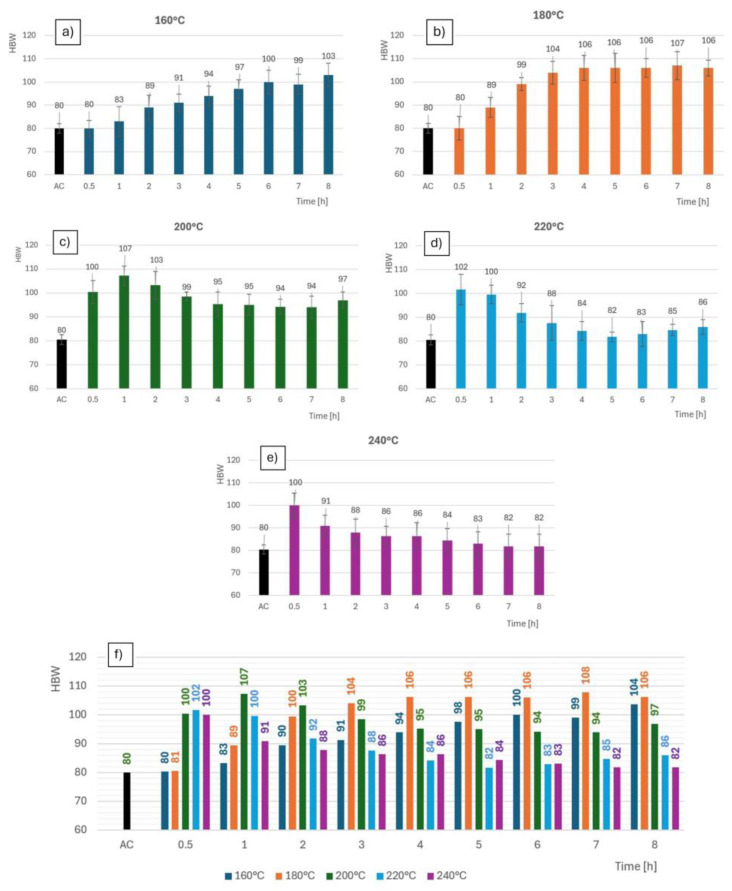
Dependence of hardness on time; for samples (**a**) 160 °C; (**b**) 180 °C; (**c**) 200 °C; (**d**) 220 °C; (**e**) 240 °C; (**f**) all samples comparison.

The values in Table 3 are the average of a minimum of seven measurements for all tested thermal regimes. Figure 10 represents the hardness of each testing mode. At lower heat temperatures, the onset of the processes was slower, which was reflected by a longer heat treatment time to reach the maximum values. The rate of hardness accumulation was shown to increase with increasing temperatures. However, it cannot be said that the rate of the process increased proportionally with increasing temperatures. On the contrary, even with a small change in temperature, the nature of the curve could change dramatically, as was observed with the change from 180 °C to 200 °C, where there was a significant increase in speed and a reduction in time.

### 3.3. Mechanical Properties

**Figure 11:**
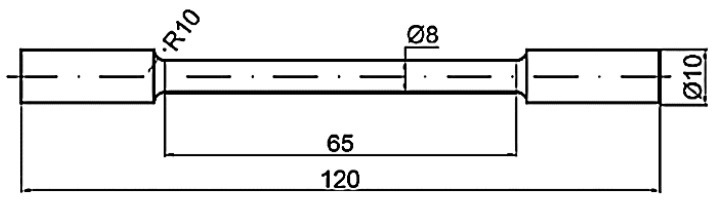
Test sample drawing.

**Figure 12:**
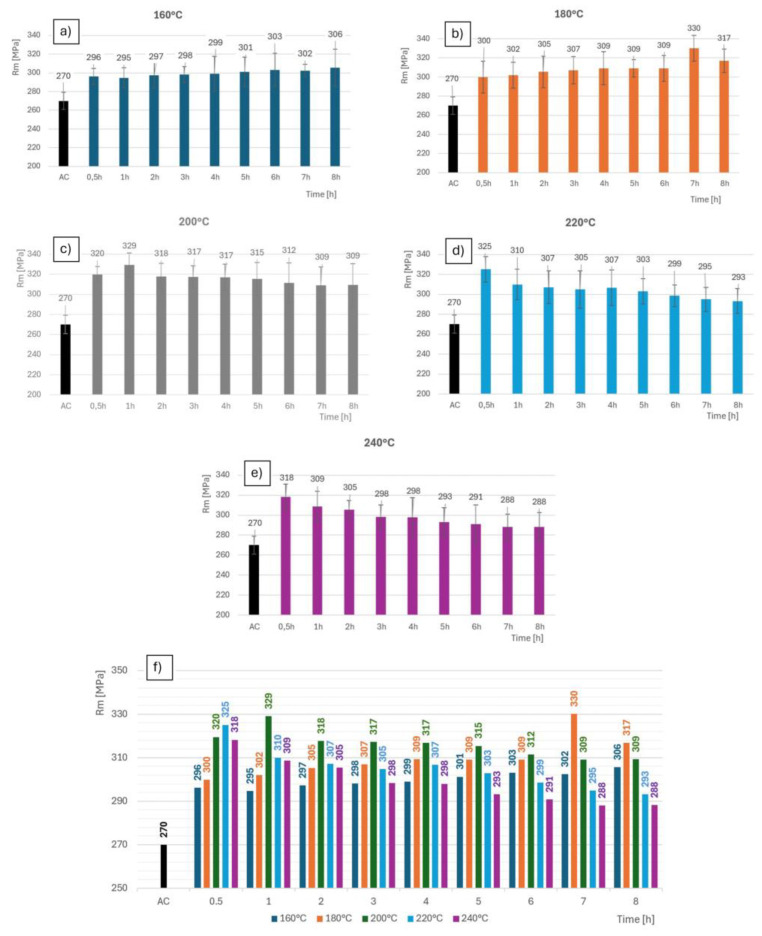
Strength limits for different heat treatment modes; for samples (**a**) 160 °C; (**b**) 180 °C; (**c**) 200 °C; (**d**) 220 °C; (**e**) 240 °C; (**f**) all samples comparison.

**Figure 13:**
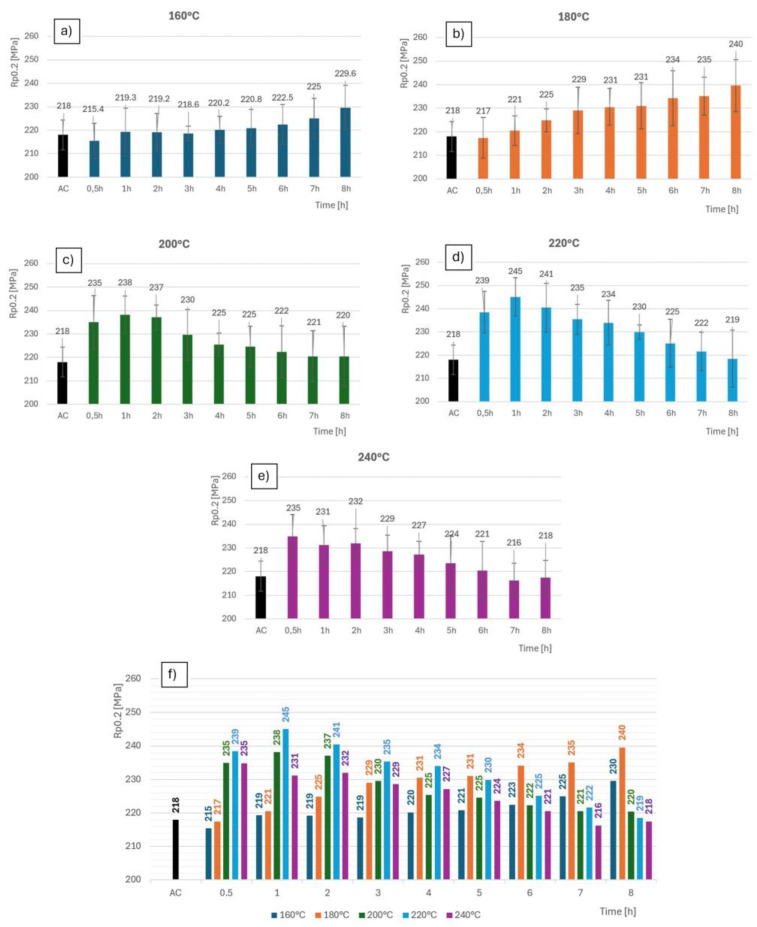
Yield strengths for different heat treatment modes; for samples (**a**) 160 °C; (**b**) 180 °C; (**c**) 200 °C; (**d**) 220 °C; (**e**) 240 °C; (**f**) all samples comparison.

**Figure 14:**
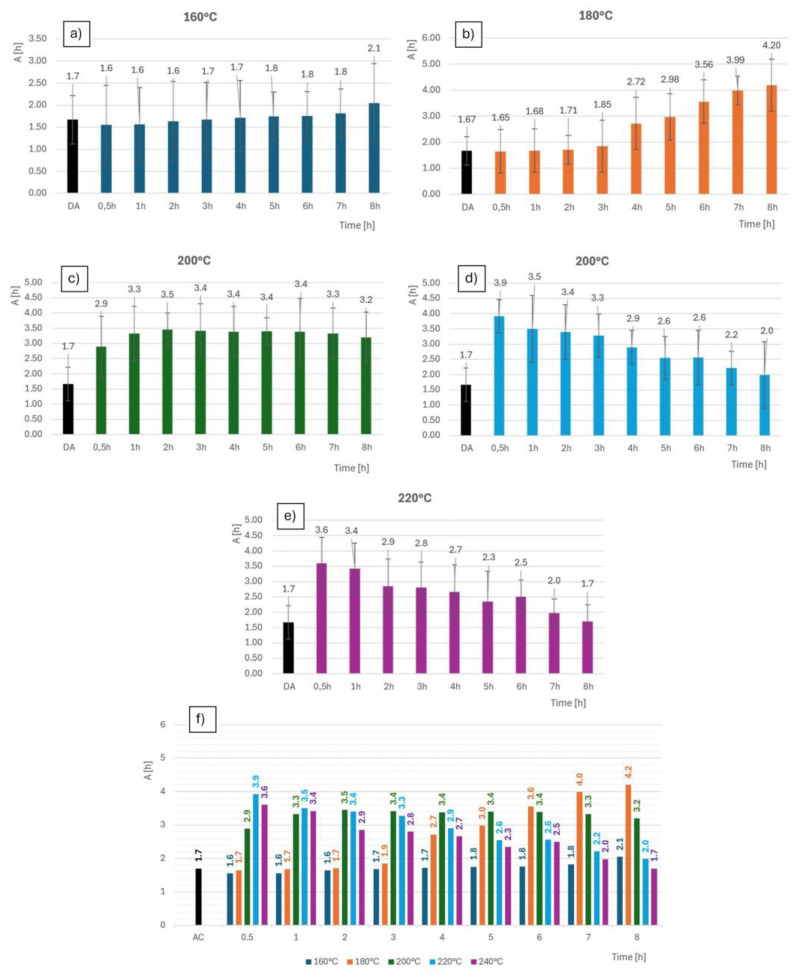
Ductility; for samples (**a**) 160 °C; (**b**) 180 °C; (**c**) 200 °C; (**d**) 220 °C; (**e**) 240 °C; (**f**) all samples comparison.

The mechanical characteristics of the specimens were determined by static tensile testing, which is classified as a short-term destructive test in terms of the duration of the loading. The test was carried out according to EN ISO 6892-1 [33] using a 50 kN Inspekt desk-type universal tear tester (Figure 11). The tear-off machine has a nominal load of 50 kN and achieves a loading rate in the range of 0.0005–250 mm/min, with the upper limit of the inter-range being reached when the nominal load is reached. The test results of the single individual experimental variants were averaged and processed in Figure 12, Figure 13 and Figure 14. For each experimental variant, a set of test round bars with a shank diameter of 8 mm was fabricated. The schematic diagram of the tensile test bars is shown in Figure 11.

#### 3.3.1. Strength Limit Rm [MPa]

The mechanical properties were evaluated by comparing the tensile tests. A minimum of five specimens were torn from each thermal regime. The results in the graphs are always their average values. By processing the results into a graphical dependence, it was clearly demonstrated that there were significant differences in terms of the ultimate strength evaluation Rm [MPa]. The Rm value of 270 MPa shows the alloy after the solution treatment (i.e., the initial condition). The greatest increase in strength was presented by the heat treatment at 180 °C for 7 h, showing an increase of 60 MPa compared with the initial condition. The average strength values are shown in Figure 12.

Interestingly, a similar strength was obtained with a higher temperature regime and shorter processing time: 200 °C for 1 h. Comparable values can also be seen at 220 °C after 30 min (325 MPa). In Table 4, the numerical values are expressed.

#### 3.3.2. Yield Strength Rp0.2 [MPa]

The effect of artificial aging was also observed when the conventional yield strength Rp0.2 [MPa] was evaluated. The experimental specimens presented growth in Rp0.2 [MPa]. The results from the measurements are presented in Table 5.

The samples processed at 160 °C and 180 °C had slower increases in yield stress compared with those processed at higher temperatures. The onset of curing was quite similar at both temperatures but only initially. After one hour, the effect of the higher temperature became apparent, and the Rp0.2 values started to differ significantly in favor of the samples treated at 180 °C. The samples reached their maximum measured strength values at 3–4 h, with an average value of 240–245 MPa at 220 °C and 239 MPa at 180 °C, but only after 10 h. With a longer residence time in the furnace, the material started to age. A closer look at the results is presented in Figure 13. The initial condition after the solution treatment was 218 MPa.

#### 3.3.3. Ductility

The gravity A [%] of the individual experimental samples significantly decreased due to heat treatment compared with the initial state after the solution treatment. The standard which prescribes the tensile test is STN EN ISO 6892-1. The principle of the test is static loading, where the dependence of the deformation of the test bar on the applied load is recorded. Tensile tests were carried out on the specimens before heat treatment, after solution treatment and after artificial aging. The greatest increase in ductility was for the 180 °C for 7 h specimen, which also exhibited increased hardness, the ultimate strength and conventional yield strength. The greatest decrease in ductility was found for the sample treated at 16 °C for 30 min. The average values from the measured ductility results are plotted in Figure 14. The ductility after solution treatment was 1.67%. The experimental variations in the temperature regimes showed wide variability not only in ductility but also in other mechanical properties.

Increasing the temperature above 200 °C resulted in a significantly faster increase in all measured mechanical properties. However, the maximum measured values were reached after only 1 h at temperatures above 200 °C. After exceeding 1 h, overaging occurred, and hardening occurred for up to 3 h. From this point on, the hardness decreased more moderately, even increasing in the last 2 h. The hardness values for the different temperature regimes are given in Table 6. For the samples processed above 200 °C, the aging process was so rapid that the increase in mechanical properties occurred only during the first half hour of the cycle. After three hours, the mechanical properties started to decrease. The measured maximum values after 30 min were, on average, 101 HBW at 220 °C. From this first measurement, it was not yet possible to assess whether the values were at their maxima at this time or whether the so-called overaging already started to occur in this time interval. It was assumed that the maximum hardness was reached in this time interval and, at the same time, the hardness started to decrease in this time interval. This assumption was based on the lower measured hardness values compared with those measured for samples which were processed at lower temperatures with a clear maximum. This assumption was particularly true for the samples processed at 240 °C, where the difference was significant.

## 4. Discussion

From the results obtained, it can be concluded that temperature and time have a significant effect on the rate of change of mechanical properties and on the maximum hardness reached. Heat treatment is used to change the shape of silicon, which will ensure better mechanical properties. The temperature influences the rate of artificial aging through the dynamics of diffusion processes which ensure the breakdown of the supersaturated solid solution. Furthermore, we can assume from the results obtained that the residence time of the samples in the furnace influenced the maximum achievable values of the mechanical properties. At lower temperatures, the increase in hardness and mechanical properties was slower and smoother. This was precisely due to the lower dynamics of the diffusion processes. By contrast, the diffusion processes accelerated with increasing temperatures, causing a sharp and rapid increase in microhardness. The temperature can therefore be used to vary the speed of the artificial aging process, which affects the heat treatment cycle time.

The heat treatment time depends on the temperature and therefore on the dynamics of the diffusion processes. A longer heat treatment time, in combination with the correct temperature, has a positive effect on the mechanical properties which the alloy can achieve [34]. We again based our results on measured values, where we obtained the highest hardness among all the samples for a 5 h cycle and 180 °C temperature. As the time shortened and hence the temperature increased, the maximum values we achieved decreased. The same was true for the temperature of 160 °C, where the maximum hardness was reached in the same time of 5 h, but its value was lower. A suitable combination of temperature and time is always important, as their different combinations affect the resulting mechanical properties of the aluminum alloy.

The effect of solution treatment resulted in spheroidization of the eutectic silicon, and the α phase manifested as rounded grains. Artificial aging caused a change in the structure. The morphology was visibly finer, but there was no marked difference between the samples. Although there was not much difference in the given microstructure images, the mechanical property measurements confirmed that the different temperatures and times of artificial aging had a great influence on the hardness and strength properties of the AlSi7Cu0.5Mg alloy. It is assumed that the hardness and strength were reached in the region of coherent precipitate formation, possibly at the beginning of the deposition of the partial coherent θ′ phase [35,36,37]. The long heat treatment process eliminated the stable θ′ phase (Al_2_Cu), resulting in a decrease in the mechanical properties. This was especially true for the samples processed above 200 °C.

## 5. Conclusions

This paper analyzed five heat treatment regimes of artificial aging of AlSi7Cu0.5Mg alloy graded from 30 min to 8 h, and based on the results, the following can be concluded:

- The highest measured hardness was achieved in the temperature regime of 180 °C at 7 h, 160 °C at 30 min.
- The best strength limit (Rm 330.1 MPa) is valid for a temperature regime of 180 °C at 7 h, and the lowest (Rm 293.2 MPa) is valid at 220 °C for 8 h.
- The highest value of the yield strength (Rp0.2 [MPa]) was 245.1 MPa at 220 °C for 1 h, and the lowest (Rp0.2 = 215.4 MPa) was valid at 160 °C for 1 h.
- The yield was highest at 180 °C for 8 h (4.2%) and lowest (1.55%) at 160 °C for 30 min.

Increasing the temperature also increased the cure rate, but at the same time, artificial aging at temperatures above 200 °C has been shown to reduce the maximum curing effect. Temperatures below 180 °C also reduced the maximum curing effect. Thus, it is ideal to choose a compromise between time and temperature to achieve the maximum effect.

## Figures and Tables

**Table 1:** Casting chemical composition.

Element	Si	Fe	Cu	Mn	Mg	Ti	Al
w.%	7.849	0.141	0.580	0.063	0.308	0.142	Ssas.

**Table 2:** Mechanical properties of AlSi7Cu0.5Mg.

Casting Process	Heat Treatment	Rm [MPa]	Re 0.2 [MPa]	A [%]	HB [HBW]
Into the mold	T6	320	250	4	100

**Table 3:** HBW 5/250/10, where 5 is the ball diameter, 250 is the contact force and 10 is the test duration in seconds.

HT [°C]	Highest HBW Value	Lowest HBW Value
160	103.6/8 h	80.3/30 min
180	107.8/7 h	80.5/30 min
200	107.2/1 h	94/7 h
220	101.6/30 min	81.7/5 h
240	100/30 min	81.8/7 h

**Table 4:** Highest and lowest measured strength limit Rm [MPa] values.

HT [°C]	Highest Value of MPa	Lowest Value of MPa
160	305.6/8 h	294.6/1 h
180	330.1/7 h	299.8/30 min
200	329/1 h	309/7 h
220	325.1/30 min	293.2/8 h
240	318.2/30 min	288/7 h

**Table 5:** Yield strength Rp0.2 [MPa] the highest and lowest measured values.

HT [°C]	Highest Value of MPa	Lowest Value of MPa
160	229.6/8 h	215.4/1 h
180	239.6/8 h	217.4/30 min
200	238.5/1 h	220.4/8 h
220	245.1/1 h	218.5/8 h
240	234.9/30 min	216.3/7 h

**Table 6:** Ductility’s [%] highest and lowest measured values.

HT [°C]	Highest % Value	Lowest % Value
160	2.05/8 h	1.55/30 min
180	4.2/8 h	1.65/30 min
200	3.45/2 h	2.89/30 min
220	3.92/30 min	1.99/8 h
240	3.6/30 min	1.7/8 h

## Data Availability

Data available on request. The data presented in this study are available on request from the corresponding author.
